# DNAJB9 Fibrillary Glomerulonephritis Following Rituximab-Based Therapy: A Case of Temporary Renal Recovery

**DOI:** 10.7759/cureus.85841

**Published:** 2025-06-12

**Authors:** Arij Ouanjine, Fatma Fendri, Elodie Miquelestorena-Standley, Remy Kerdraon, Manon Dekeyser

**Affiliations:** 1 Department of Nephrology, Centre Hospitalier Universitaire d'Orléans, Orleans, FRA; 2 Department of Pathology, Centre Hospitalier Universitaire de Tours, Tours, FRA; 3 Inserm U1327 ISCHEMIA "Membrane signalling and inflammation in reperfusion injuries", Tours University, Tours, FRA; 4 Department of Pathology, Centre Hospitalier Universitaire d'Orléans, Orleans, FRA; 5 LI2RSO “Orléans Interdisciplinary Laboratory for Innovation and Research in Health”, Orleans University, Orleans, FRA

**Keywords:** acute kidney injury, dnajb9, fibrillary glomerulonephritis, kidney biopsy, rituximab

## Abstract

A 63-year-old man, with no relevant history, developed acute kidney injury with an elevated serum creatinine level of 314 µmol/L associated with hypertension, a nephrotic syndrome, without hematuria. Kidney biopsy revealed a glomerular-specific deposition of DnaJ homolog subfamily B member 9 (DNAJB9). Fibrillary glomerulonephritis was diagnosed. The patient received corticosteroids, rituximab (1 g at day 1 and day 15; 1 g at month 6), and nephroprotection. Kidney dysfunction initially worsened (creatinine 513 µmol/L), and peritoneal dialysis was initiated. Partial renal function recovery was observed after the rituximab-maintenance dose, allowing dialysis discontinuation for one year (October 2023 to September 2024). In some studies, rituximab-based therapy was associated with stabilization of disease progression. As observed in our case, it could be considered on a case-by-case basis, with a possible benefit for partial treatment response and time-limited disease control.

## Introduction

Fibrillary glomerulonephritis (FGN) is an uncommon immune-complex glomerulopathy characterized by randomly oriented, non-amyloid fibrils along the glomerular basement membrane. It accounts for less than 1% of native kidney biopsies [1]. The median age at diagnosis is typically in the sixth decade, with no clear gender predominance [2]. DnaJ homolog subfamily B member 9 (DNAJB9) immunostaining provides a highly sensitive and specific diagnostic biomarker that complements electron microscopy [3]. DNAJB9 is a co-chaperone protein normally involved in intracellular protein folding; in FGN, it aberrantly accumulates within the glomeruli and co-localizes with the fibrillary deposits, serving as both a diagnostic marker and a potential contributor to pathogenesis [4]. Patients typically present with nephrotic-range proteinuria and variably decreased renal function. The renal prognosis is generally poor, with more than 50% of patients requiring dialysis within a few years of diagnosis, despite immunosuppressive therapy [1,5]. While no standardized treatment exists, rituximab is used empirically [1]. Rituximab is a monoclonal antibody that targets CD20-positive B cells, which are believed to play a central role in the formation of pathogenic immune complexes in FGN, leading to B-cell depletion. Although clinical responses to rituximab are inconsistent, its use is based on this immunopathogenic rationale and supported by small observational studies [2,5,6]. This report describes the case of a 63-year-old man with DNAJB9-positive FGN who achieved a one-year withdrawal from peritoneal dialysis following rituximab therapy, illustrating both the therapeutic challenges and the potential for partial renal recovery.

## Case presentation

**Figure 1:**
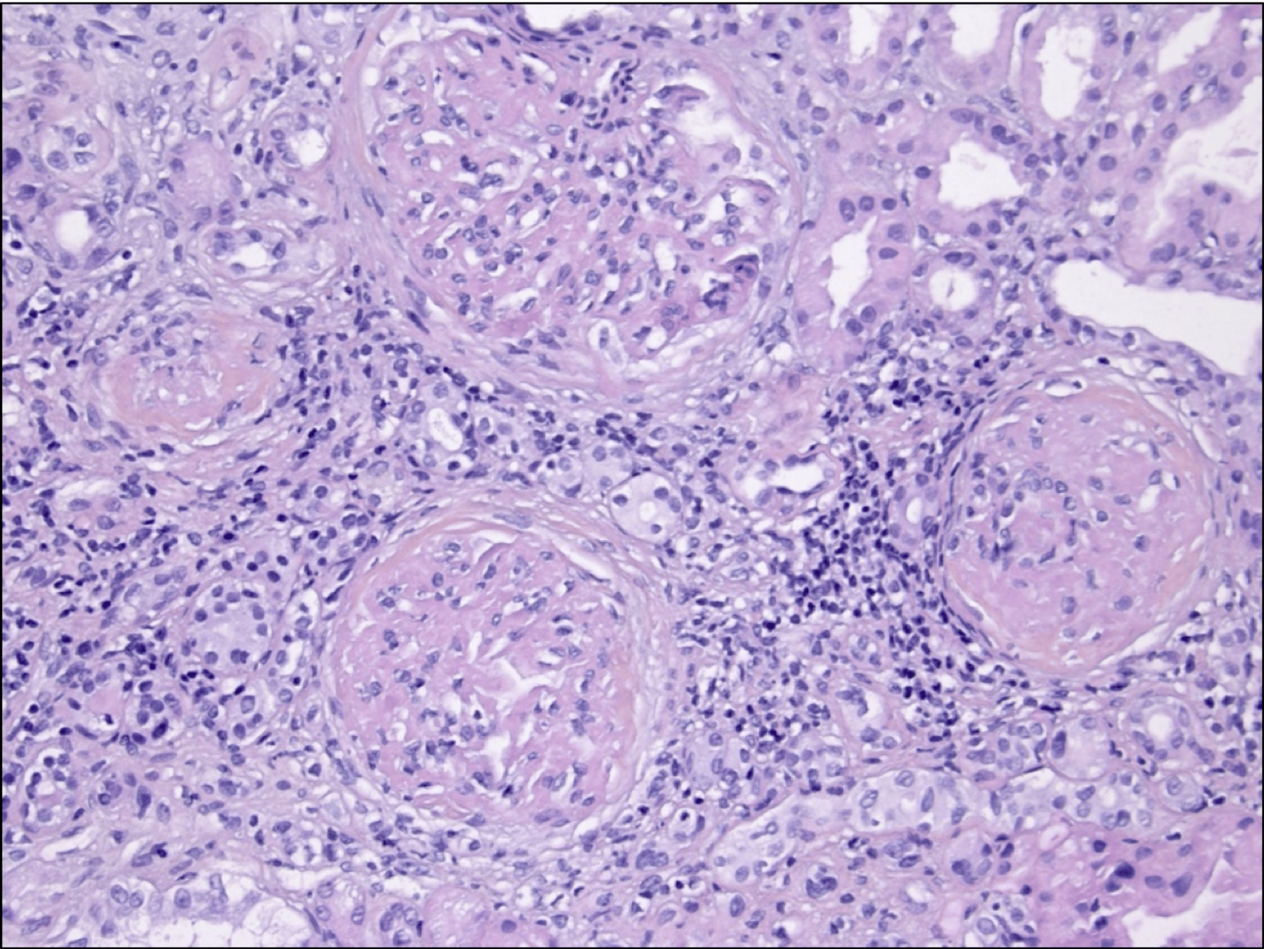
Renal biopsy disclosing glomerular deposition of immunoglobulin Light microscopy of renal biopsy stained with hematoxylin-eosin-saffron (original magnification ×200) showing expanded mesangium and thickened capillary walls with eosinophilic material consistent with fibrillary deposits. These histologic changes raised suspicion for fibrillary glomerulonephritis, later confirmed by immunohistochemistry.

**Figure 2:**
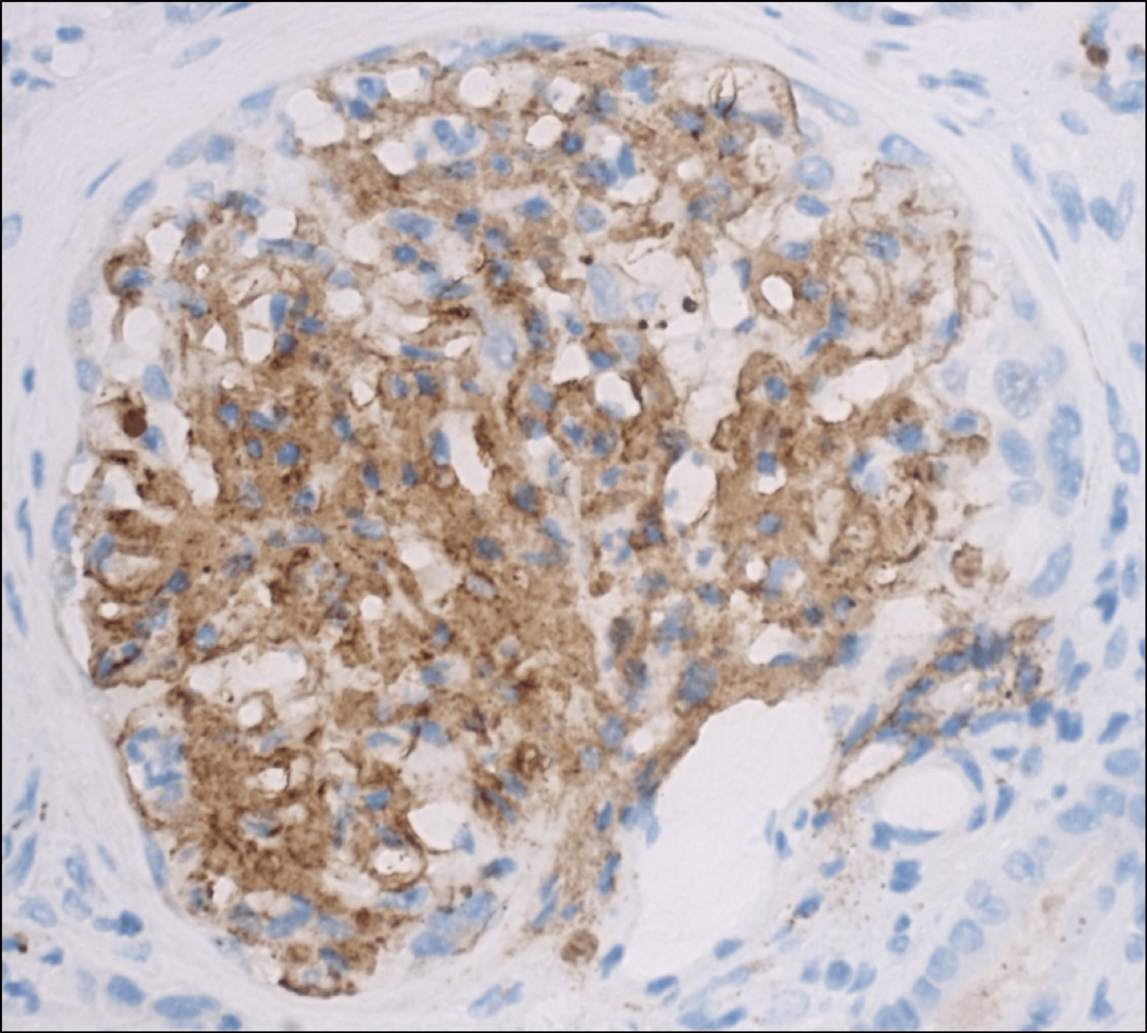
Renal biopsy disclosing glomerular-specific deposition of DnaJ homolog subfamily B member 9 (DNAJB9) antibody Immunohistochemistry of renal biopsy using anti-DNAJB9 antibody (Sigma Life Science, The Woodlands, TX) at 400x magnification reveals strong, glomerular-specific cytoplasmic positivity. DNAJB9 positivity is a highly sensitive and specific marker for FGN, aiding in differentiating it from other glomerular diseases with fibrillar deposits.

In February 2023, a 63-year-old man with a history of testicular embryonal carcinoma treated by orchidectomy and chemotherapy in 2010 was admitted to our nephrology department for acute kidney injury. Physical examination revealed moderate grade II hypertension with blood pressure 170/69 mmHg. Serum creatinine level was increased (314 µmol/L versus 150 µmol/L at baseline). Urinary analysis showed proteinuria of 6.49 g/day without hematuria or leukocyturia. The haptoglobin and complement levels were normal. Autoimmune and infectious tests were negative, notably anti-phospholipase A2-receptor, anti-neutrophil cytoplasmic antibody, cryoglobulinemia, and hepatitis C serological test. Albuminemia was 19.8 g/L. Serum protein electrophoresis with immune fixation studies disclosed a heterogeneous restriction in gamma-globulins, without a monoclonal immunoglobulin spike. Kappa and lambda free light chains were 85 and 45 mg/L, respectively, with an average ratio for kidney failure. A whole-body 18F-FDG PET (18F-fluorodeoxyglucose positron emission tomography) scan was performed and did not reveal any hypermetabolic lesions suggestive of malignancy. Relevant laboratory findings, including detailed serum, urine, and immunological parameters, are summarized in Table 1. The kidney biopsy revealed 15 glomeruli. Hematoxylin-eosin-saffron (HES) staining demonstrated eosinophilic deposits within the mesangium and along the glomerular capillary walls, consistent with fibrillary material, without evidence of endocapillary proliferation or cellular crescents (Figure 1). Periodic acid-Schiff (PAS) staining showed positive deposits, which appeared green on trichrome stain, and were negative on silver and Congo red stains, thereby excluding amyloidosis. Mild tubular atrophy and interstitial fibrosis were also present. Immunofluorescence studies revealed diffuse granular deposits of IgG (predominantly IgG1), C3, C1q, and C4d along the glomerular capillary walls and mesangium. The detection of both kappa and lambda light chains indicated a polytypic immune complex deposition. Immunostaining for DNAJB9 showed strong glomerular-specific positivity (Figure 2). No glomerular staining was observed for anti-PLA2R or other autoantibodies. Although electron microscopy was not performed, the presence of pseudo-amyloid features on histology in combination with DNAJB9 positivity strongly supports the diagnosis of DNAJB9-associated FGN.

**Table 1:** Summary of laboratory results eGFR, estimated glomerular filtration rate; CRP, C-reactive protein; HIV, human immunodeficiency virus; HBV, hepatitis B virus; HCV, hepatitis C virus; ANA, antinuclear antibodies; ANCA, antineutrophil cytoplasmic antibodies; hCG, human chorionic gonadotropin; PSA, prostate-specific antigen.

Parameter	Value at Admission	Unit	Reference Range
Serum creatinine	314	µmol/L	53-106
Urea	10.3	mmol/L	2.5-7.5
eGFR	22	mL/min/1.73 m²	>60
Proteinuria (spot)	6.88	g/g	<0.20
Serum albumin	19.8	g/L	35-55
Serum sodium	138	mmol/L	135-145
Serum potassium	5.9	mmol/L	3.5-5.1
Serum bicarbonate	17	mmol/L	22-28
Urine sediment	12 WBCs, 7 RBCs	/mm³	<10 mm³
Leukocytes	6	G/L	4-10
Hemoglobin	11.8	g/dL	13.5-17.5
Platelets	357	G/L	150-400
CRP	5	mg/L	<5
Serologies (HIV, HBV, HCV)	Negative	-	Negative
Serum protein electrophoresis and immunofixation	No monoclonal immunoglobulin spike	-	-
Free light chains λ	45	mg/L	5.7-26.3
Free light chains κ	85	mg/L	3.3-19.4
κ/λ ratio	1.88	-	0.37-3.1
C3	1.23	g/L	0.9-1.8
C4	0.25	g/L	0.1-0.4
Cryoglobulins	Negative	-	Negative
ANA	Negative	-	Negative
ANCA	Negative	-	Negative
Anti-PLA2R	Negative	-	Negative
Alpha-fetoprotein	3	ng/mL	<10
hCG	1.0	mIU/mL	<5
PSA	1.02	ng/mL	<4

**Figure 3:**
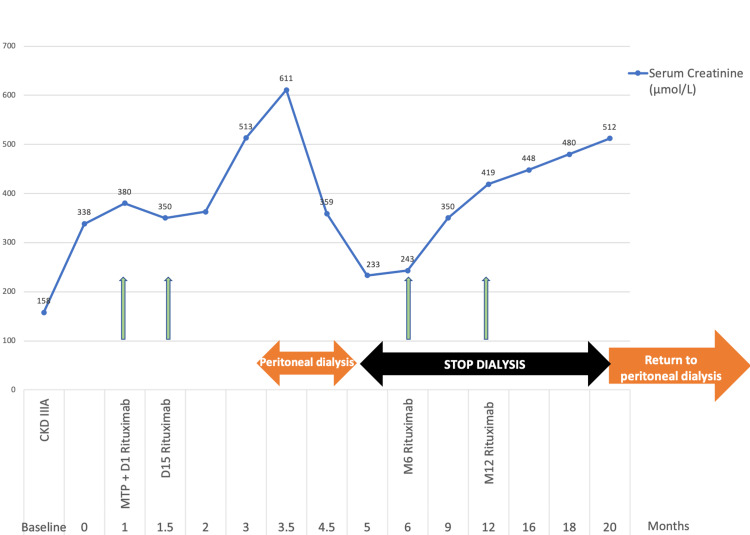
Patient’s serum creatinine and clinical evolution under rituximab therapy demonstrating the dialysis discontinuation for one year. The graph shows initial deterioration leading to peritoneal dialysis, followed by partial renal recovery after a maintenance rituximab dose, enabling dialysis discontinuation for 13 months. The chart illustrates the transient therapeutic response and progressive renal decline characteristic of advanced FGN. FGN, fibrillary glomerulonephritis; CKD, chronic kidney disease; MTP, methylprednisolone.

Based on this diagnosis, the patient was initiated on corticosteroid therapy at 1 mg/kg/day, in combination with rituximab-based immunosuppression (1 g on days 1 and 15), consistent with prior reports despite the absence of a standardized treatment protocol [6,7]. An angiotensin-converting enzyme (ACE) inhibitor was also introduced for nephroprotection. The initial outcome was marked by a severe impairment of renal function, with a serum creatinine level of 473 µmol/L and the initiation of peritoneal dialysis. After the rituximab-maintenance dose (1 g at month 6), we observed a partial renal function recovery, allowing peritoneal dialysis withdrawal for one year, from October 2023 to September 2024. Peritoneal dialysis had to be resumed in October 2024 due to the unfavorable progression of his chronic kidney disease (Figure 3).

## Discussion

FGN is a rare glomerular disease, found in less than 1% of native kidney biopsies [1]. As in our case, precise diagnosis relies on histopathological assessment, particularly with highly specific DNAJB9 immunohistochemical staining. Electron microscopy is important for confirming fibrillary deposits, but it is not always available in clinical practice. FGN is characterized by glomerular deposition of randomly oriented, Congo red-negative fibrils of 12-24 nm diameter. The fibrils are located predominantly in the mesangium within the glomerular capillary basement membrane, and much more rarely in tubular basement membranes. Immunofluorescence demonstrates polyclonal IgG (IgG4 and IgG1 subclasses mainly) and C3 deposits. Rare cases of monoclonal FGN are also described [2].

Differential diagnoses should always be carefully considered and systematically ruled out. Renal amyloidosis is excluded by negative Congo red staining and the absence of apple-green birefringence under polarized light [3]. Immunotactoid glomerulopathy is characterized by organized microtubular structures on electron microscopy [3,4]. Diabetic fibrillosis is easily excluded based on the clinical context. DNAJB9 is not detected in renal amyloidosis, immunotactoid glomerulopathy, or diabetic glomerulopathy with fibrillosis, further confirming its diagnostic specificity [4]. Consequently, many experts propose renaming the entity “DNAJB9-associated FGN.”

Recently, the discovery of DNAJB9 as a sensitive and specific tissue biomarker of FGN has revolutionized FGN diagnosis. DNAJB9 is a co-chaperone of heat shock protein 70 involved in the endoplasmic reticulum protein-folding process. Although its role in the pathogenesis of FGN remains unknown, the accumulation of DNAJB9 in FGN deposits suggests that the disease could be driven by unfolded and misfolded proteins [3]. DNAJB9 could serve as an antigen triggering an IgG4-dominant autoimmune response, despite the absence of identified circulating DNAJB9 autoantibodies [8]. A second theory is that, rather than being an autoantigen, DNAJB9 adheres secondarily to misfolded IgG molecules while recognizing aggregation-prone motifs. This may partly explain the weak response of FGN to immunosuppressive therapy. Anti-DNAJB9 monoclonal antibodies are being explored as potential targeted treatments, but have yet to demonstrate efficacy [8].

While FGN predominantly affects the kidney, approximately one-third of patients have associated malignancies or autoimmune diseases, necessitating a comprehensive evaluation [1]. Previously classified as a monoclonal gammopathy of renal significance, recent studies have shown that most FGN cases are polyclonal, differentiating it from other deposition diseases like amyloidosis or cryoglobulinemia [9].

FGN has a poor prognosis, with over 50% of patients progressing to end-stage renal disease [1]. Rituximab remains the most studied immunosuppressive agent for FGN. In a 12-month prospective pilot study involving 11 patients, rituximab led to a 56% reduction in proteinuria, though changes in serum creatinine were not statistically significant. Only three patients (27%) achieved partial remission [6]. Two larger retrospective series of 66 and 27 patients, respectively, reported response rates (partial or complete) ranging between 13% and 30% [1,5]. A third retrospective study, including 12 patients, noted a 72% response rate, defined as stable renal function over three years, but used broader remission criteria [7]. This discrepancy may reflect longer follow-up and more lenient definitions of response. In one published case, combination therapy with rituximab and an SGLT2 inhibitor led to an 85% reduction in proteinuria at one year [10]. Our patient did not receive SGLT2 inhibitors due to severely reduced estimated glomerular filtration rate (eGFR).

Monitoring CD19+ B-cell depletion after rituximab infusion is commonly used to confirm pharmacodynamic activity and to rule out underdosing, especially in patients with nephrotic syndrome, where urinary loss of rituximab and altered recycling may reduce its efficacy [11-13]. However, while B-cell monitoring can verify depletion, it does not reliably correlate with clinical improvement. Several studies, including the MENTOR trial in membranous nephropathy, have shown that CD19+ cell levels do not predict remission or renal recovery [6,12,13]. Similar observations have been reported in lupus nephritis and other autoimmune diseases, highlighting that clinical assessment remains essential for evaluating treatment response [11]. Otherwise, rituximab is well tolerated, with infusion reactions being the most common adverse events [6,7]. 

Overall, rituximab showed modest benefit and disease stabilization in FGN. Given the absence of standardized treatment protocols or remission criteria, rituximab should be considered on a case-by-case basis, particularly in progressive disease [1,5]. Future prospective studies with standardized endpoints and longer follow-up are needed to better define its role in FGN management.

## Conclusions

FGN carries a poor renal prognosis: half of the patients progress to end-stage kidney disease within four years, and a large subgroup deteriorates within months of diagnosis despite immunosuppression. Early detection of high-risk features and predictors of rapid progression (nephrotic-range proteinuria, low baseline eGFR, extensive fibrosis, aggressive histologic pattern) is crucial for patient counseling and for considering rituximab therapy on a case-by-case basis. Rituximab may offer partial treatment response and time-limited disease control, as observed in the literature. Our case emphasizes the potential benefits of rituximab while also underscoring the importance of setting appropriate expectations regarding outcomes and the need for ongoing monitoring.
